# Draft Genome Sequence of Microcystis aeruginosa NIES-87, a Bloom-Forming Cyanobacterium from Lake Kasumigaura, Japan

**DOI:** 10.1128/genomeA.01596-17

**Published:** 2018-02-22

**Authors:** Haruyo Yamaguchi, Shigekatsu Suzuki, Masanobu Kawachi

**Affiliations:** a Center for Environmental Biology and Ecosystem Studies, National Institute for Environmental Studies, Ibaraki, Japan

## Abstract

Microcystis aeruginosa is a problematic cyanobacterium in freshwater lakes distributed worldwide. Here, we report the draft genome sequence of M. aeruginosa NIES-87, isolated from Lake Kasumigaura, Japan. The genome is approximately 4.9 Mb in size, with an average G+C content of 42.9% and 4,355 predicted protein-coding genes.

## GENOME ANNOUNCEMENT

Microcystis aeruginosa is one of the most harmful bloom-forming cyanobacteria in freshwater (1). It is a unicellular colony-forming cyanobacterium distributed worldwide in eutrophic freshwater environments. During summer, Microcystis blooms result in serious environmental problems, such as bad odor releases, bottom-layer anoxia, and production of hepatotoxic cyanotoxins called microcystins. Microcystins are the only cyanotoxins for which the World Health Organization has set drinking and recreational water standards (2). M. aeruginosa NIES-87 was isolated from Lake Kasumigaura, Japan. This strain is available from the Microbial Culture Collection at the National Institute for Environmental Studies, Japan (http://mcc.nies.go.jp). It is an axenic culture and does not produce microcystin; however, it releases a bad odor, the origin of which is unknown. This strain also produces an antialgal peptide kasumigamide (3). A recent multilocus phylogenetic study suggested that M. aeruginosa is genetically divided into at least eight clades (groups A to G and X) (4). In that study, the phylogenetic position of NIES-87 was not well resolved. Tominaga et al. (5) reported that this strain harbors two plasmids with sizes of 2.3 and 5 kb. This strain has been widely used in various studies; however, to date, whole-genome sequencing of the strain has not been conducted. Here, we report the draft genome sequence of M. aeruginosa NIES-87. This information would be useful for unveiling genome evolution in M. aeruginosa.

DNA extraction was performed using 10 ml of NIES-87 axenic culture using the DNeasy plant minikit (Qiagen), following the manufacturer’s instructions. Subsequently, DNA was fragmented to approximately 550 bp using Covaris M220. Genomic libraries of paired ends and mate pairs were constructed using the TruSeq Nano DNA library prep kit (Illumina) and the Nextera mate pair library preparation kit (Illumina), respectively. Whole-genome sequencing was performed using the MiSeq platform (Illumina) employing the 600-cycle MiSeq version 3 reagent kit. The resulting paired-end and mate pair reads were 1,576,274 and 1,403,390 in number, respectively. Low-quality reads/bases were filtered using Trimmomatic version 0.36 (6), and *de novo* assembly was performed using SPAdes version 3.7.1 (7). The resulting genome comprised 206 contigs of 4,947,287 bp. The average genome coverage of the paired-end reads was 95.3×. The maximum contig length was 652,566 bp. Genome annotation was performed using the DDBJ Fast Annotation and Submission Tool (DFAST) (8). The genome comprised 4,355 predicted protein-coding sequences, one set of rRNAs, and 41 tRNAs. The G+C content of the genome was 42.9%. The genome was scanned using antiSMASH (9), and microviridin and other secondary metabolite gene clusters were predicted. The 16S rRNA sequence of NIES-87 was subjected to a BLAST search, and the top hits obtained were those of M. aeruginosa NIES-2481 and NIES-2549 (99.8% identity each). We used OrthoFinder (10) to identify orthologous genes among the three genomes, and 56.9% and 57.1% of the genes in NIES-2481 and NIES-2549, respectively, were identified as orthologous to those of NIES-87.

### Accession number(s).

This whole-genome shotgun project has been deposited in GenBank under the accession no. BFAC01000001 to BFAC01000206.
